# Molecular Cloning of a cDNA Encoding for *Taenia solium* TATA-Box Binding Protein 1 (TsTBP1) and Study of Its Interactions with the TATA-Box of Actin 5 and Typical 2-Cys Peroxiredoxin Genes

**DOI:** 10.1371/journal.pone.0141818

**Published:** 2015-11-03

**Authors:** Oscar Rodríguez-Lima, Ponciano García-Gutierrez, Lucía Jiménez, Ángel Zarain-Herzberg, Roberto Lazzarini, Abraham Landa

**Affiliations:** 1 Departamento de Microbiología y Parasitología, Facultad de Medicina, Universidad Nacional Autónoma de México, México D.F., México; 2 Departamento de Química, Universidad Autónoma Metropolitana–Iztapalapa, México D.F., México; 3 Departamento de Bioquímica, Facultad de Medicina, Universidad Nacional Autónoma de México, México D.F., México; 4 Departamento de Biología Experimental, Universidad Autónoma Metropolitana–Iztapalapa, México D.F., México; Queensland Institute of Medical Research, AUSTRALIA

## Abstract

TATA-box binding protein (TBP) is an essential regulatory transcription factor for the TATA-box and TATA-box-less gene promoters. We report the cloning and characterization of a full-length cDNA that encodes a *Taenia solium* TATA-box binding protein 1 (TsTBP1). Deduced amino acid composition from its nucleotide sequence revealed that encodes a protein of 238 residues with a predicted molecular weight of 26.7 kDa, and a theoretical pI of 10.6. The NH_2_-terminal domain shows no conservation when compared with to pig and human TBP1s. However, it shows high conservation in size and amino acid identity with taeniids TBP1s. In contrast, the TsTBP1 COOH-terminal domain is highly conserved among organisms, and contains the amino acids involved in interactions with the TATA-box, as well as with TFIIA and TFIIB. *In silico* TsTBP1 modeling reveals that the COOH-terminal domain forms the classical saddle structure of the TBP family, with one α-helix at the end, not present in pig and human. Native TsTBP1 was detected in *T*. *solium* cysticerci´s nuclear extract by western blot using rabbit antibodies generated against two synthetic peptides located in the NH_2_ and COOH-terminal domains of TsTBP1. These antibodies, through immunofluorescence technique, identified the TBP1 in the nucleus of cells that form the bladder wall of cysticerci of *Taenia crassiceps*, an organism close related to *T*. *solium*. Electrophoretic mobility shift assays using nuclear extracts from *T*. *solium* cysticerci and antibodies against the NH_2_-terminal domain of TsTBP1 showed the interaction of native TsTBP1 with the TATA-box present in *T*. *solium* actin 5 (pAT5) and 2-Cys peroxiredoxin (Ts2-CysPrx) gene promoters; in contrast, when antibodies against the anti-COOH-terminal domain of TsTBP1 were used, they inhibited the binding of TsTBP1 to the TATA-box of the pAT5 promoter gene.

## Introduction

Transcription is the process to generate RNA from a gene, and it is carried out by different RNA polymerases. It is known that some genes possess the TATA-box motif in its core promoters [1]. In them, the TATA-box binding protein (TBP) interacts directly with DNA though of this motif, which is typically located at -25 to -35 base pair (bp) relative to the Transcription Start Site (TSS) and has the consensus sequence TATA(A/T)A(A/T) [2, 3]. TBP is an important protein that, together with other general transcription factors (GTFs), forms the pre-initiation complex (PIC) allowing the polymerase to bind the promoter genes and initiate the transcription process. TBP is a component of SL1, TFIID, and TFIIIB complexes, which are used by RNA polymerases I, II and III, respectively [4–6].

The majority of genes transcribed by all three RNA polymerases lack TATA-box in their promoters; nevertheless, TBP interacts with TBP-associated factors (TAFs) to form the PIC for the RNA Polymerase binding [7–9].

TBPs structure consists of an NH_2_-terminal domain (NH_2_-ter), necessary for species-specific transcription factor binding, with variable amino acid residues and is non-conserved among species, and a COOH-terminal domain (COOH-ter) that is highly conserved and is formed by ~180 residues [6, 10]. This domain presents the classical saddle structure formed by 10-anti parallel β-strands that form a concave domain that contains all the amino acids implicated in DNA binding and four amphipathic α-helices that form the convex domain, which has the amino acids needed for the interaction with some GTF´s [11].

Although information exists about gene core promoter sequences, genomes, and transcriptomes of *Echinoccocus ganulosus*, *E*. *multilocularis*, *Taenia crassiceps*, and *T*. *solium*, little is known about the role that TBPs and others transcription factors are playing in the transcription process in the *Taeniidae* family [12–20]. Therefore, the aim of this project was to clone and characterize the cDNA encoding a TATA-box binding protein 1 of *T*. *solium* (TsTBP1) and to study the interaction of this protein with the TATA-box in the core promoter of actin 5 (pAT5) and typical 2-Cys peroxiredoxin (Ts2-CysPrx) genes of *T*. *solium*.

## Material and Methods

### Biological materials

Cysticerci from *T*. *solium* were dissected from skeletal muscle of a naturally infected swine, acquired of Temixco, Morelos, Mexico. Located in geographical coordinates 18°51′16″ North latitude and 99°13′38″ West longitude. For infected swine identification, several backyard-breeding animals were inspected by visual analysis and tongue palpation looking for sub-epithelial cysticerci. Infected swine were selected and slaughtered for the purposes of this study. *T*. *crassiceps* WFU strain cysticerci were obtained from experimentally infected mice, briefly, six weeks old female BALB/cJ mice were infected with 10 WFU strain *T*. *crassiceps* cysticerci in peritoneal cavity using a 20G needle and euthanized 90 days after cysticerci inoculation [21]. The mice were maintained in groups of six mice with water and hormone-additives-pesticides free food *at libitum*. They were monitored every day, all mice presented good health until sacrifice at 90 days. Cysticerci obtained were washed 4 times with sterile ice-cold phosphate-buffered saline (PBS: 137 mM NaCl, 2.7 mM KCl, 8.1 mM Na_2_HPO_4_, and 1.47 mM KH_2_PO_4_, pH 7.2), and used for experiments.

### Ethics statement

Swine were slaughtered by desensitization and posterior bleeding, according to the Official Mexican Norm: NOM-033-ZOO-1995 for humanitarian sacrifice of domestic and wild animals and the procedure inspected by veterinarian staff. All mice were reproduced and maintained in a pathogen-free and controlled environmental conditions (20 ± 2 °C temperature and 55 ± 5% humidity) and 12 h light/dark cycle at Facultad de Medicina, UNAM animal care facilities. Additionally, animals were euthanized by using i.v. pentobarbital (210 mg/kg), according to the Official Mexican Norm: NOM-062-ZOO-1999 for production, care and use of laboratory animals. All protocols were in strict accordance with the Guide for the Care and Use of Laboratory Animals of the NIH, USA. The research protocol was approved by Research and Ethic Committee of the Facultad de Medicina, Universidad Nacional Autónoma de México (007–2012).

### *Taenia solium* TBP1 cDNA cloning

TsTBP1 probe was generated using the Super Script One Step RT-PCR kit (Invitrogen, Carlsbag, CA), using 1 μg of *T*. *solium* cysticerci´s total RNA and primers designed for two well-conserved sequences in TBPs (TBP5´: RNAEYNP and TBP3´: YEPELFP). Program for cDNA synthesis was 45°C for 30 min, and for PCR amplification, 30 cycles of 94°C for 1 min, 52°C for 30 sec, 72°C for 1min and a cycle for final extension of 72°C for 15 min. The fragment obtained was purified, cloned into pCRII vector (Invitrogen), and sequenced on an automated DNA sequencer ABI Prism model 373 (Applied Biosystems, Grand Island, NY). The probe obtained was used to isolate a full clone of TsTBP1 by screening 45,000 clones from the *T*. *solium* adult cDNA library constructed in λZAP II and carried out as previously described [22]. Inserts of positive clones were amplified by PCR, cloned into pCRII, and sequenced as before. Bioinformatics analysis such as the translation of nucleotide sequence and multiple alignments were carried out using the PCGENE and Clustal X programs.

### Molecular modeling

The structure for the deduced sequence of TsTBP1 was modeled with the *Homology Model* implemented in MOE software package (*Molecular Operating Environment* 2013.08; Chemical Computing Group Inc., Montreal, Canada) using the human TFIIA/TBP/DNA complex X-ray structure as template (PDB ID: 1NVP). The potential force field was Amber12:EHT with a reaction field treatment of solvation electrostatics. Ten models were built as a result of the permutational selection of different loop candidates and side chain rotamers. The best intermediate model, according to the GB/VI function score implemented in MOE, was subjected to further optimal energy minimization in explicit solvent using a Root-mean-square deviation (RMSD) gradient equal to 0.01. Prior, *Protonate 3D* implemented in MOE was used to assign the protonation state of ionizing residues. The geometric quality of the final homology model was verified by Ramachandran plot. The Adaptative Poisson-Boltzmann Solver (APBS) program [23] was used within PyMOL to display the results of the calculations as an electrostatic potential molecular surface. The ionic strength was set to 100 mM of NaCl.

### Antibody production

Two different peptides were synthesized, one at the beginning of the NH_2_-ter and designed as pTsTBP1-N (MQPTPINQLVSVVGSYAAPSSTQAHSRPPYTPNTPG), and the other at the end of the COOH-ter and designed as pTsTBP1-C (VRDEIYQAFNNIYPILKNFMKLDSDKSGLHQPALTG). Rabbit polyclonal antibodies were produced by four subcutaneous immunizations every 2 weeks, using 100 μg of each synthetic peptide mixed with saponin (10 μg). The rabbits were bled 7 days after the last immunization, and the obtained sera were frozen at -20°C. The immunoglobulin G (antibodies) fraction was purified from rabbit sera by affinity chromatography with protein G-agarose (Sigma-Aldrich, ST. Louis, MO), titer and specificity of antibodies were determined by ELISA and western blot.

### *Taenia solium* nuclear extract

*Taenia solium* cysticerci (10 g) were homogenized in buffer A (20 mM HEPES, 20 mM KCl, 1.5 mM MgCl_2_, 25% Glycerol, 0.5% Nonidet P-40, 0.2 mM EDTA, 1 μM Pepstatin, 0.6 μM Leupeptin, 0.2 mM PMSF, 0.5 mM DTT) with Ultra Turrax T8 (IKA, Wilmington, NC). The suspension was incubated 10 min and centrifuged at 10,000xg for 20 min. The pellet was suspended in 4 ml of buffer A, added to a tube containing 10 ml Ficoll solution (5.7%), and centrifuged at 3,300xg for 15 min. The pellet was resuspended in 1 ml of buffer B (20 mM HEPES, 1.2 mM KCl, 1.5 mM MgCl_2_, 0.2 mM EDTA, 25% Glycerol, 1 μM Pepstatin, 0.6 μM Leupeptin, 0.2 mM PMSF, 0.5 mM DTT) and gently shaken for 45 min. The suspension was centrifuged at 14,000xg for 30 min; the supernatant was quantified by the Bradford method, aliquoted and stored at -20°C until use. All steps of the protocol were carried out at 4°C. Nuclear extract (2.5 μg/mm of gel) integrity was determined in a 10% SDS-PAGE with 2-mercaptoethanol and stained with Coomassie blue.

### TsTBP1 identification by western blot and immunofluorescence

For western blot, 5 μg of nuclear extract per mm of gel were transferred to PVDF membranes. Membranes were incubated with rabbit anti-pTsTBP1-N, anti-pTsTBP1-C antibodies, and normal rabbit IgG at 1:100 dilution, washed with PBS-0.3% Tween and incubated with peroxidase-conjugated anti-rabbit IgG. Bound antibodies were revealed with 3,3´-diaminobenzidine and 1% H_2_O_2_.

Cysticerci from *T*. *crassiceps* were embedded in Tissue-Freezing Medium (Triangle Biomedical Science, Durham, NC), frozen in liquid nitrogen, and stored at -70°C. Frozen sections of 6 to 8 μm thick were prepared and fixed with 4% paraformaldehyde in PBS. Samples were permeabilized with 0.01% (v/v) Triton-X 100 for 30 min, and blocked with 3% (w/v) BSA in PBS for 30 min; sections were incubated overnight with anti-pTsTBP1-N, anti-histone H1-sc-393530 antibodies (Santa Cruz Biotechnology, Dallas, TX), and normal mouse and rabbit IgG were used as negative controls (all antibodies were diluted 1:100). Sections were rinsed three times with PBS and incubated 60 min at room temperature (rt) with Alexa 568-conjugated anti-mouse IgG and Alexa 488-conjugated anti-rabbit IgG (diluted 1:200 in PBS-3% BSA, Life Technologies, Grand Island, NY). Sections were rinsed three times with PBS and incubated 5 min at rt with 4′,6-diamidino-2-phenylindole (DAPI). Sections were rinsed as before and mounted on a glycerol-PBS solution (9:1). Single plane images were obtained with a confocal microscope LSM-META-Zeiss Axioplan 2. Co-localization analysis was performed using the ZEN 2010 program version 6.0 (Carl Zeiss, Pleasanton, CA).

### Electrophoretic mobility shift assay (EMSA)

To generate complementary double-stranded DNA oligonucleotide (dsDNA) probes, both oligonucleotides were mixed at 1:1 molar ratio, heated to 95°C for 5 min and gradually cooled to rt. dsDNA probes were labeled with [γ-^32^P]ATP (Perkin Elmer, Boston, MA) using T4 polynucleotide kinase. Binding reactions were performed by pre-incubating at rt: 17.5 fmol of each labeled probe, 1 μg of poly(dI-dC) and 10 μg of nuclear extract in binding buffer (20% glycerol; 2.5 mM EDTA; 5 mM MgCl_2_; 250 mM NaCl; 50 mM Tris-HCl; 2.5 mM DTT). For competition, 25, 50 and 100-fold molar excess of unlabeled dsDNA probe was added to the binding reaction. The reactions were incubated 30 min at rt. For super-shift assay, 1 μg of anti-pTsTBP1-N antibodies were added to the reaction after adding the nuclear extract, and incubated for 30 min at rt. All reactions were finished with addition of gel-loading buffer (15% Ficoll, 0.25% Bromophenol Blue, 0.25% Xylene Cyanol in TBE). The complex formed was separated on a non-denaturing 5% polyacrylamide gel and visualized by autoradiography of the dried gel [24]. For densitometric analysis, the EMSA films were digitized using a scanner and analyzed with the ImageJ program [25]. Statistical significance was defined as two tailed Student´s t-test, P < 0.005, and results are present as percentage mean ± SD of the shifted band.

To determine, if anti-pTSTBP1-C is able to inhibit the binding of TsTBP1 to the TATA-box of the pAT5 gene promoter, EMSA was performed as described above, but using a biotin-labeled DNA probe (TATA-box). The probe was labeled with the Labeling Kit (Pierce, Grand Island, NY). Briefly, EMSA was carried out as follows: for supershift assay, 17.5 fmol of TATA-box of pAT5-biotin-labeled was incubated at rt with 1 μg of poly(dI-dC) and 10 μg of *T*. *solium* nuclear extract in binding buffer for 30 min, then, 1 μg of anti-pTsTBP1-C antibodies was added to the reaction and incubated 30 min. To inhibit the binding of TsTBP1 to TATA-box of pAT5, 10 μg of *T*. *solium* nuclear extract were incubated with 1 μg of anti-pTsTBP1-C antibodies, 1 μg of poly(dI-dC) in binding buffer for 30 min, and then, 17.5 fmol of pAT5 TATA-box biotin-labeled was added and incubated at rt for 30 min more. Additionally, normal rabbit IgG and anti-pTsTBP1-N antibodies were used instead of anti-TsTBP1-C antibodies, for supershift and inhibition reactions, as a control. Shift reaction with 17.5 fmol of TATA-box of pAT5-biotin-labeled was carried out as described above.

## Results

### Nucleotide sequence analysis of TsTBP1 cDNA

**Fig 1:**
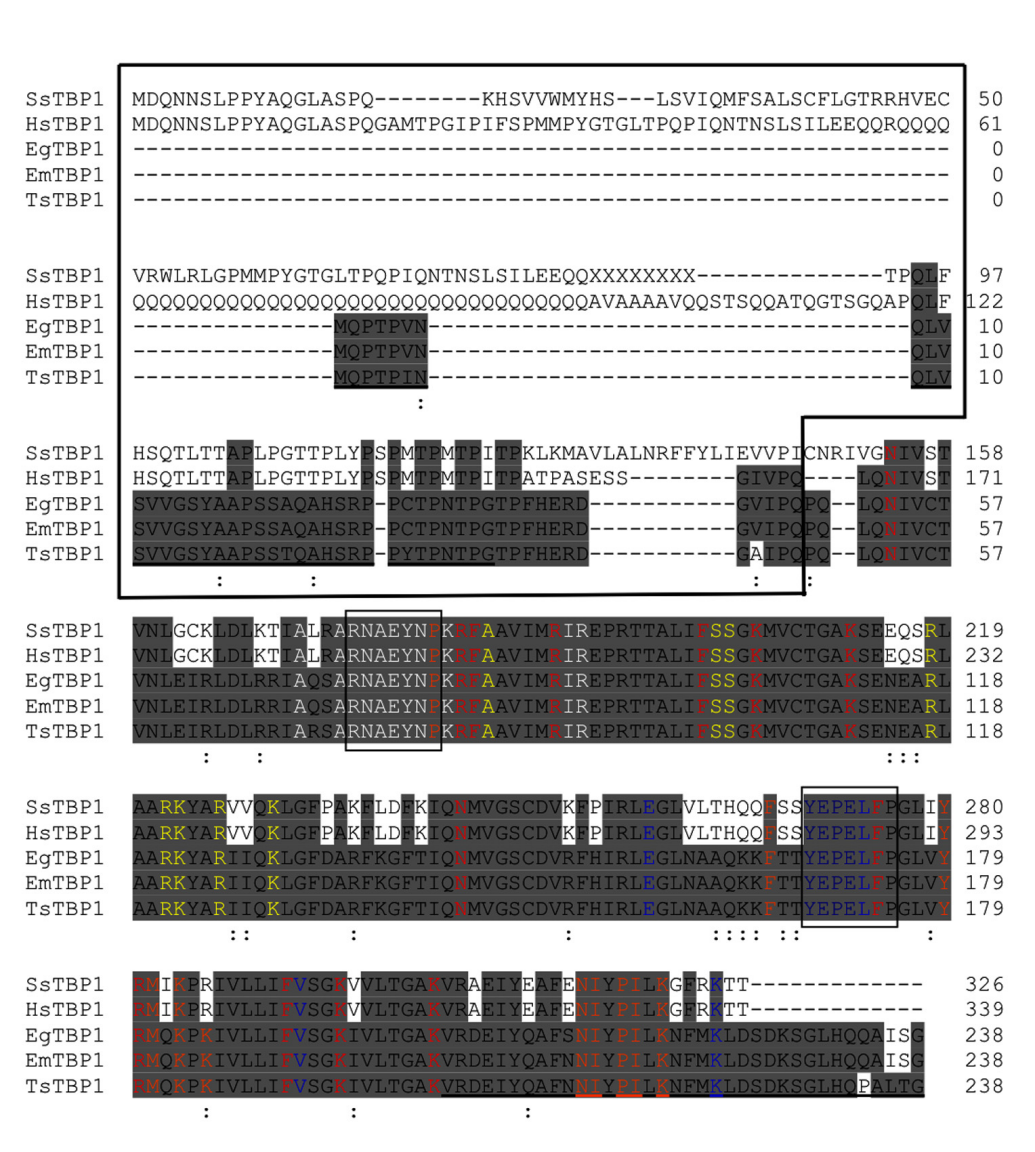
Multiple amino acid alignment of TATA-box binding protein 1 from *T*. *solium* (TsTBP1). TsTBP1 was aligned with *Sus scrofa* TBP1 (SsTBP1, GeneBank ID: XP_003361466.1), *Homo sapiens* TBP1 (HsTBP1, GeneBank ID: NP_003185.1), *E*. *granulosus* TBP1 (EgTBP1, GeneBank ID: CDS17003.1), *E*. *multilocularis* TBP1 (EmTBP1, GeneBank ID: CDJ04746.1). The NH_2_-ter is enclosed in a box, and the remaining amino acid sequence corresponds to the COOH-terminal domain (COOH-ter). Identical amino acids are highlighted in gray background. Important residues that bind TATA-box are in red letters; transcription factor II A (TFIIA) in white; transcription factor II B (TFIIB) in blue; negative cofactor 2 (NC2) in orange and TBP1-associated factor 1 (TAF1) in yellow. Amino acid sequences used to produce the TsTBP probe and the synthetic peptides pTsTBP1-N and pTsTBP1-C are in small boxes and underlined, respectively. Letter X on *S*. *scrofa* TBP1 sequence means amino acids not identified. The symbols under the amino acids indicate: (-) absence and (:) homology of amino acids.

A 306 bp probe homologous to TsTBP1 was produced through RT-PCR using total RNA from *T*. *solium* cysticerci and two degenerate oligonucleotides from well-conserved TBP motifs (see Fig 1). This cDNA probe allowed us to isolate two identical phage clones from a cDNA library of adult stage *T*. *solium*, containing the complete coding sequence for TsTBP1. The cDNA sequence spans 919 bp, with an open reading frame (ORF) from position 37 bp to 751 bp, where the start codon codes for methionine and the stop codon correspond to TAA. The clone possesses a 36 bp 5´-UTR and a 168 bp 3´-UTR, which contains a putative polyadenylation signal at position 880 bp (AGTAGA) [26] and a tail of 18 adenosines. The ORF of the obtained cDNA encodes a polypeptide of 238 amino acids, with a predicted M_r_ of 26.7 kDa and a theoretical pI of 10.6. The nucleotide sequence TsTBP1 cDNA and its deduced amino acid sequence are deposited in GenBank under the accession KR673321.

A multiple primary sequence alignment (Fig 1) shows that TsTBP1 has a high identity with TBP1 from *E*. *multilocularis* (97%, EmTBP1) and with *E*. *granulosus* (96%, EgTBP1); whereas less identity was observed with *Homo sapiens* TBP1 (64%, HsTBP1) and *Sus scrofa* TBP1 (62%, SsTBP1). The NH_2_-ter is about two thirds shorter and highly conserved among EgTBP1, EmTBP1, and TsTBP1 (92%) and less conserved in TBP1s from pig and human (31%). Moreover, its COOH-ter presents all important residues involved in the recognition of DNA (*i*.*e*., N^53^, R^82^, F^83^, R^89^, F^100^, K^104^, K^111^, N^143^, F^174^, R^180^, F^191^, K^195^, K^202^) and binding of GTFs, such as TFIIA (*i*.*e*., A^70^, A^73^, R^74^ N^75^, A^76^, E^77^, Y^78^, N^79^, K^81^, R^89^, I^90^ R^91^), TFIIB (*i*.*e*., E^157^, Y^169^, E^170^, P^171^, E^172^, L^173^, R^180^, V^192^, K^223^), NC2 (*i*.*e*., P^80^, R^82^, F^166^, Y^179^, R^180^, M^181^, K^183^, K^185^, N^213^, I^214^, P^216^, I^217^, K^219^), and TAF1 (*i*.*e*., A^70^, R^74^, N^75^, E^77^, Y^78^, A^84^, R^91^, S^101^, S^102^, R^117^, R^121^, K^122^, R^125^, K^129^) [27–35]. Likewise, a remarkable feature is that there are 13 additional residues in the taeniids that there are not present in pig and human TBP1s.

### *In silico* modeling of the TsTBP1 structure

**Fig 2:**
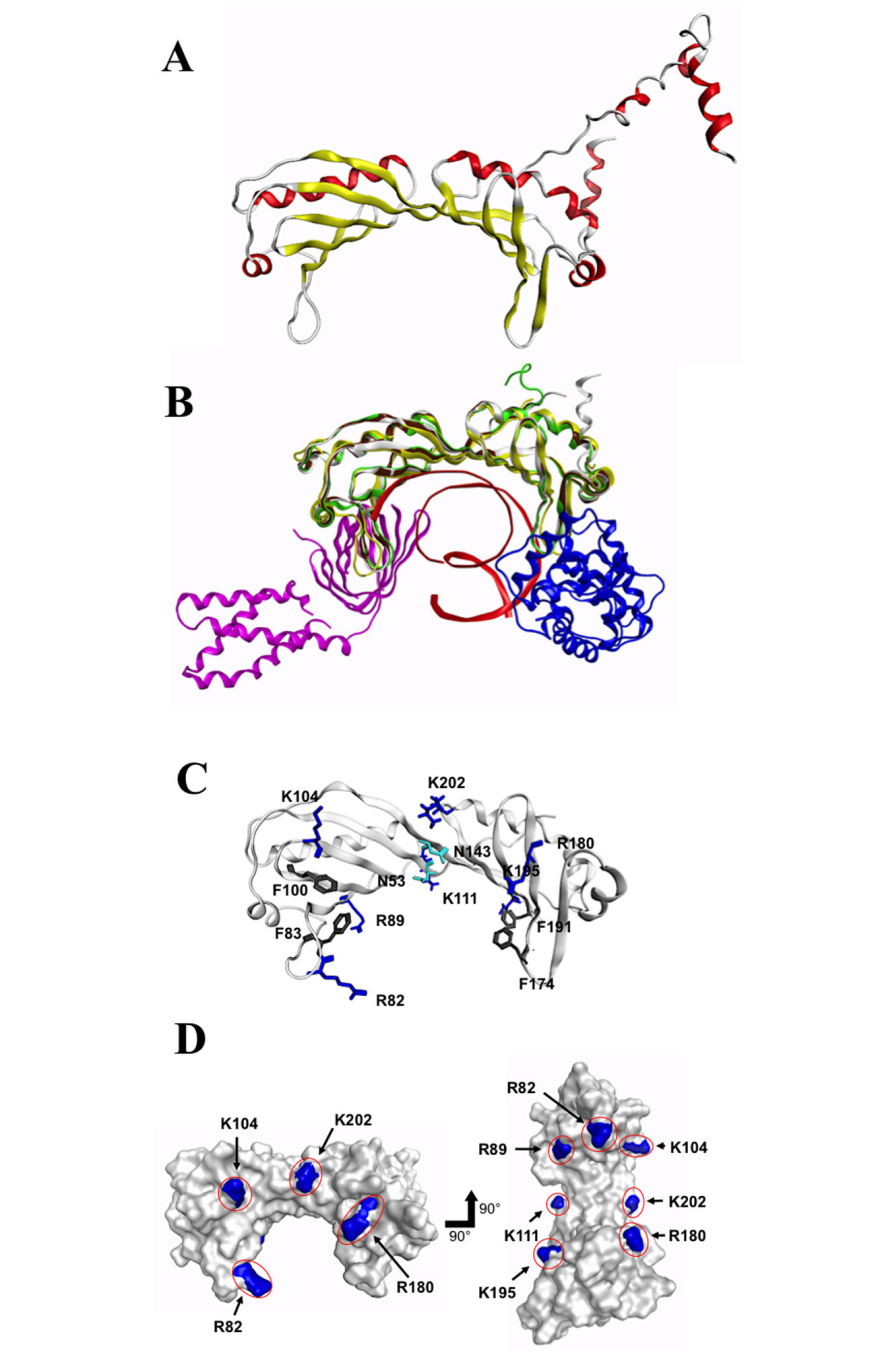
Structural analysis of TsTBP1. A) Ribbon representation of a 3D-homology model for TsTBP1 from the deduced amino acid sequence. It shows a conserved COOH-ter and non-conserved NH_2_-ter of TBP structure (β-strands are in yellow, α-helices are in red). B) Superposition of the COOH-ter of TsTBP1 model (in white) with X-ray structure of *Saccharomyces cerevisiae* TBP1 (in yellow. PDB ID 1RM1), human TFIIB/TBP/DNA complex (in blue, green, and red. PDB ID: 1VOL) and human TFIIA/TBP1 complex (in pink and brown. PDB ID 1NVP). C) Localization in the TsTBP1 model of amino acids involved in DNA recognition (in dark blue) and phosphate groups (gray and cyan). D) Solvent-accessible surface of the COOH-ter TsTBP1 model showed in front and bottom views. The blue patches show the positive density produced by the basic amino acids involved in DNA binding.

Fig 2A shows a homology model of TsTBP1 constructed according to the amino acid sequence deduced from cDNA and using the X-ray structure of the human TFIIA/TBP/DNA complex as template (PDB ID: 1NVP). To evaluate the quality of the resulting model, the Ramachandran plot was constructed, and its inspection shows that there were no amino acids in disallowed regions. The structure obtained for COOH-ter consists of two nearly identical subdomains that adopt a quasi-symmetric α/β structure, resembling a saddle. The concave underside of the saddle is highly curved, and constituted by 10-anti parallel β-strands, and the convex upper surface of the saddle consists of five α-helices, both contain important conserved amino acids for the recognition of DNA and GTFs, previously mentioned. Additionally, random coils and three small α-helices compose the NH_2_-ter of TsTBP1. Because, NH_2_-term is widely divergent in size and sequence across species [6]; moreover; its high mobility, has make difficult to determine its structure by X-ray diffraction [11, 36]. Therefore this domain was deleted for subsequent analysis. Fig 2B shows the superposition of the COOH-ter of TsTBP1 model with 3D structures of yeast TBP1 (PDB ID: 1TBP), human TFIIB/TBP/DNA complex (PDB ID: 1VOL) and human TFIIA/TBP/DNA complex (PDB ID: 1NVP) where a high grade of structure similarity is observed in the core proteins. The RMSD values (only considering C_α_) obtained were 1.32, 1.04, and 0.52 Å, respectively (Fig 2B). Fig 2B also shows the regions of interaction of TsTBP1 (white), Human TBP1 (green and brown, HsTBP1) and *Saccharomyces cerevisiae* TBP1 (yellow, ScTBP1) with TFIIA (pink structure), TFIIB (blue structure), and DNA (in red). Noteworthy, the regions of interaction are structurally conserved and fit with the crystallographic data for these GTFs and DNA. As mentioned before, all of the important residues for interaction are conserved suggesting a strong interaction. Moreover, an extra α helix at the end of TsTBP1 of the COOH-ter was identified, which is absent in HsTBP1, and ScTBP1. Fig 2C shows the TsTBP1 COOH-ter structural model revealing the distribution and localization on the concave underside of the residues involved in the interaction with DNA or TATA-box motif (Fig 1). In the TPB/DNA complex from yeast (PDB ID 1YTB), a string of lysine and arginine residues (colored dark blue) that interacts with the phosphate groups of the DNA; four phenylalanine residues (colored in black) that jam into the DNA minor groove forming the kinks that bend the DNA. There are also two symmetrical asparagine residues that form hydrogen bonds at the center. Notice that TsTBP1, a single protein chain, is composed of two symmetrical halves, and is easily seen in the pairs of phenylalanine residues and the two asparagines. Fig 2D shows the positive patches located over the conserved positive amino acids involved in DNA recognition (*i*.*e*., R^82^, R^89^, K^104^, K^111^, R^180^, K^195^, K^202^) and reveals the solvent access surface. These residues generate a positive charge density over the concave underside isosurface of the saddle, and allow a strong interaction with negatively charged phosphates in the DNA backbone.

### Identification and localization of TBP1 in cysticerci

**Fig 3:**
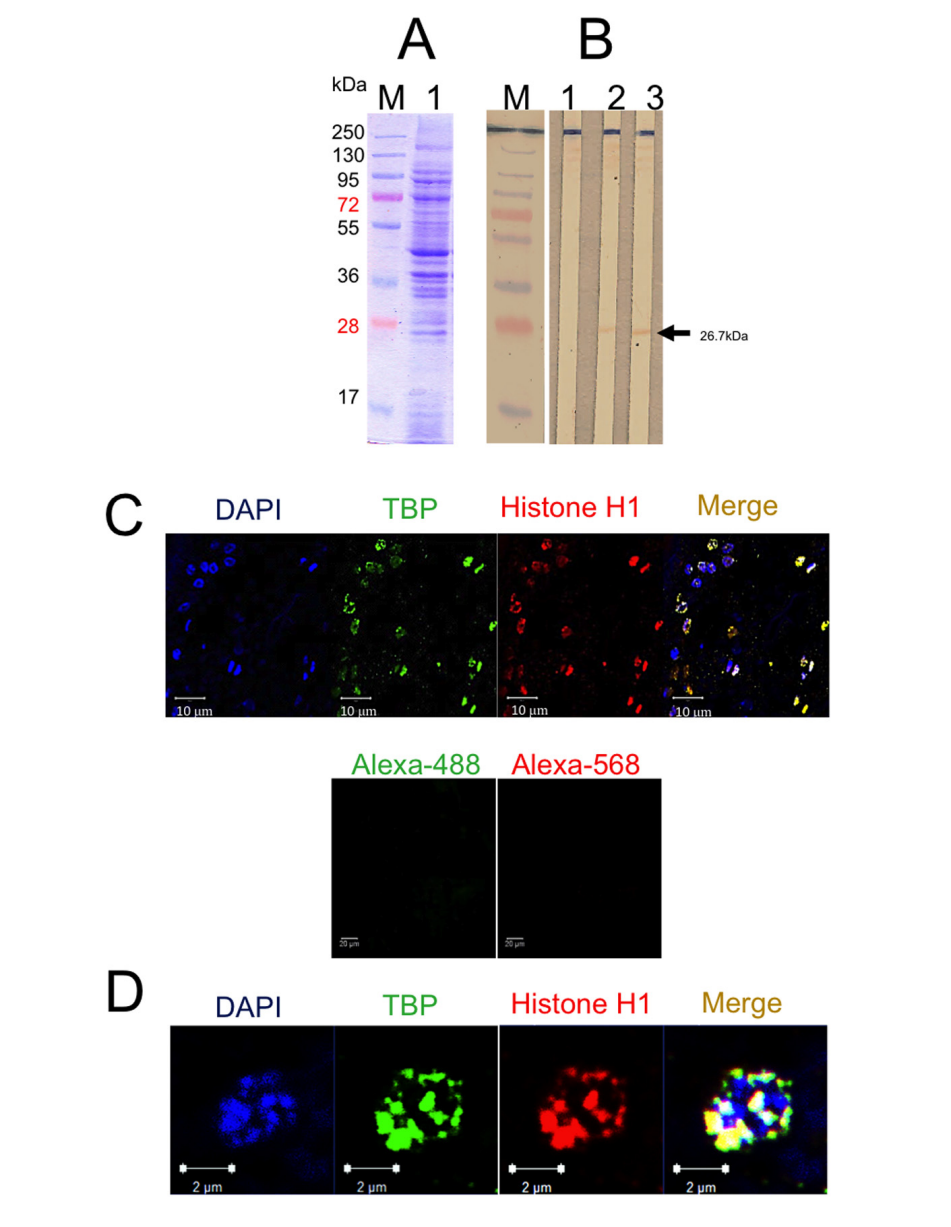
Composition of nuclear extract proteins and immunodetection of native TsTBP1. A) 10% SDS-PAGE of cysticerci *T*. *solium* nuclear extract patterns stained with Coomassie blue (lane 1). B) Western blot of TsTBP1 on *T*. *solium* nuclear extract with: normal serum IgG (lane 1), anti-pTsTBP1-N (lane 2), and anti-pTsTBP1-C antibodies (lane 3). C) Localization of TBP1 on *Taenia crassiceps* cysticerci sections by confocal microscopy with DAPI (blue), anti-histone H1 (green), anti-pTsTBP1-N antibodies (red) and merging of previous images (yellow signal). Negative control for primary and secondary antibodies, were normal mouse IgG plus anti-mouse IgG-Alexa-568 and normal rabbit IgG plus anti-rabbit IgG-Alexa-488. D) Digital amplification of a single nucleus to observe the localization of DNA (blue), histone H1 (green), and TBP1 (red), and their co-localization (yellow signal).

*Taenia solium* nuclear extract protein integrity was observed in an SDS-PAGE stained with Coomassie blue, showing proteins in a range between 10 to 250 kDa (Fig 3A, lane 1). Western blot assay showed that anti-pTsTBP1-N and anti-pTsTBP1-C antibodies recognized a band of ~26 kDa (native TsTBP1) in *T*. *solium* nuclear extract, Fig 3B, lanes 2 and 3, respectively. In contrast, no bands were recognized when normal rabbit IgGs were used as negative control (Fig 3B, lane 1). Confocal microscopy with DAPI, anti-pTsTBP1-C and anti-histone H1 antibodies revealed the presence of DNA (blue), TsTBP1 (green), and histone H1 (red) in form of specks in the nucleus of the cells that form the vesicular wall of *T*. *crassiceps* cysticerci, moreover no signal was observed when normal IgG from mouse or rabbit and second antibodies (anti-mouse IgG-Alexa-568, and anti-rabbit IgG-Alexa-488) were used as controls (Fig 3C). Merging of the image (Fig 3C) and its amplification (Fig 3D) show the co-localization of DNA, TBP1, and histone H1, as yellow specks, and a faint blue signal (DAPI) present where no signal to histone H1 and TBP1 was seen (Merging of Fig 3D). Finally, the faint signal observed in the cytoplasm of some cells could be due to the anti-histone H1 and anti-pTsTBP1-C antibodies that may recognize these molecules during their transport to the nucleus after being translated in the ribosomes. Analysis of the fluorescence on the xyz planes showed that the TBP1 and histone H1 signal are inside the nucleus (S1 Fig), and the quantification of fluorescence shows a 100:79 ratio for histone H1 and TsTBP1, respectively (S2 Fig), not statistical significance was observed.

### TsTBP1 interactions with the TATA-box motif

**Fig 4:**
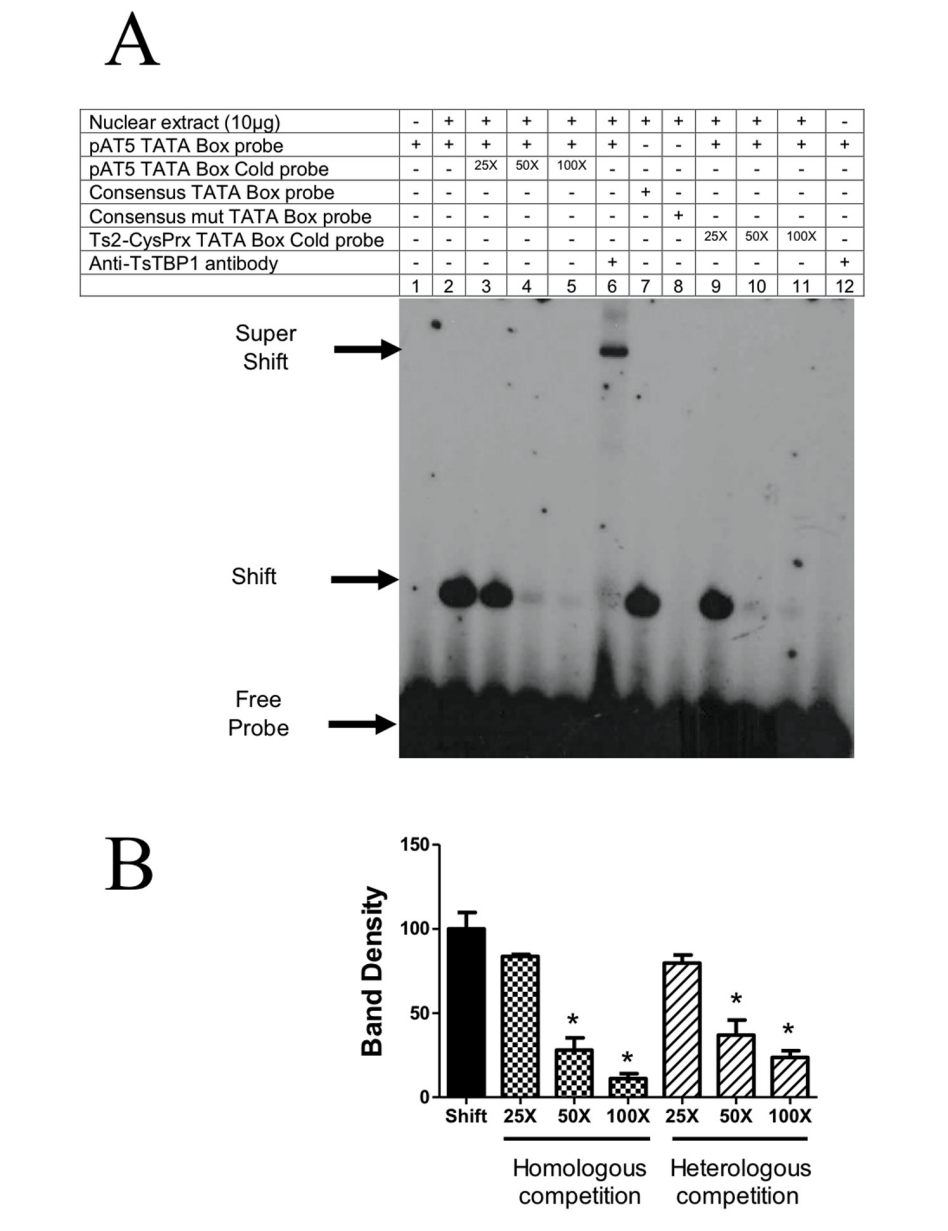
Electrophoretic mobility shift assay showing the interaction of wild type TsTBP1 pAT5 TATA-box probe. A) Lane 1: Labeled dsDNA-^32^P probe without nuclear extract; lane 2: TsTBP1-pAT5 TATA-box interaction with *T*. *solium* nuclear extract; lanes 3, 4, and 5: competence with pAT5 TATA-box cold probe in a molar excess of 25X, 50X, and 100X, respectively; lane 6: super-shift interaction using anti-pTsTBP1-N; lane 7: consensus TATA-box probe interaction with *T*. *solium* nuclear extract (used as positive control); lane 8: consensus mutated TATA-box probe interaction with nuclear extract (used as negative control); lane 9, 10 and 11: cross-competence with Ts2-CysPrx TATA-box cold probe in a molar excess of 25X, 50X, and 100X, respectively; lane 12: anti-TsTBP1-N antibody without *T*. *solium* nuclear extract (negative control). Shifted, super-shifted bands and the free-labeled dsDNA probe, are indicated by arrows. B) Densitometric analysis shows a decrease on the intensity of shifted bands in homologous and heterologous competition. Results are present as percentage mean ± SD of the shifted band in lane 2 (P < 0.005).

**Fig 5:**
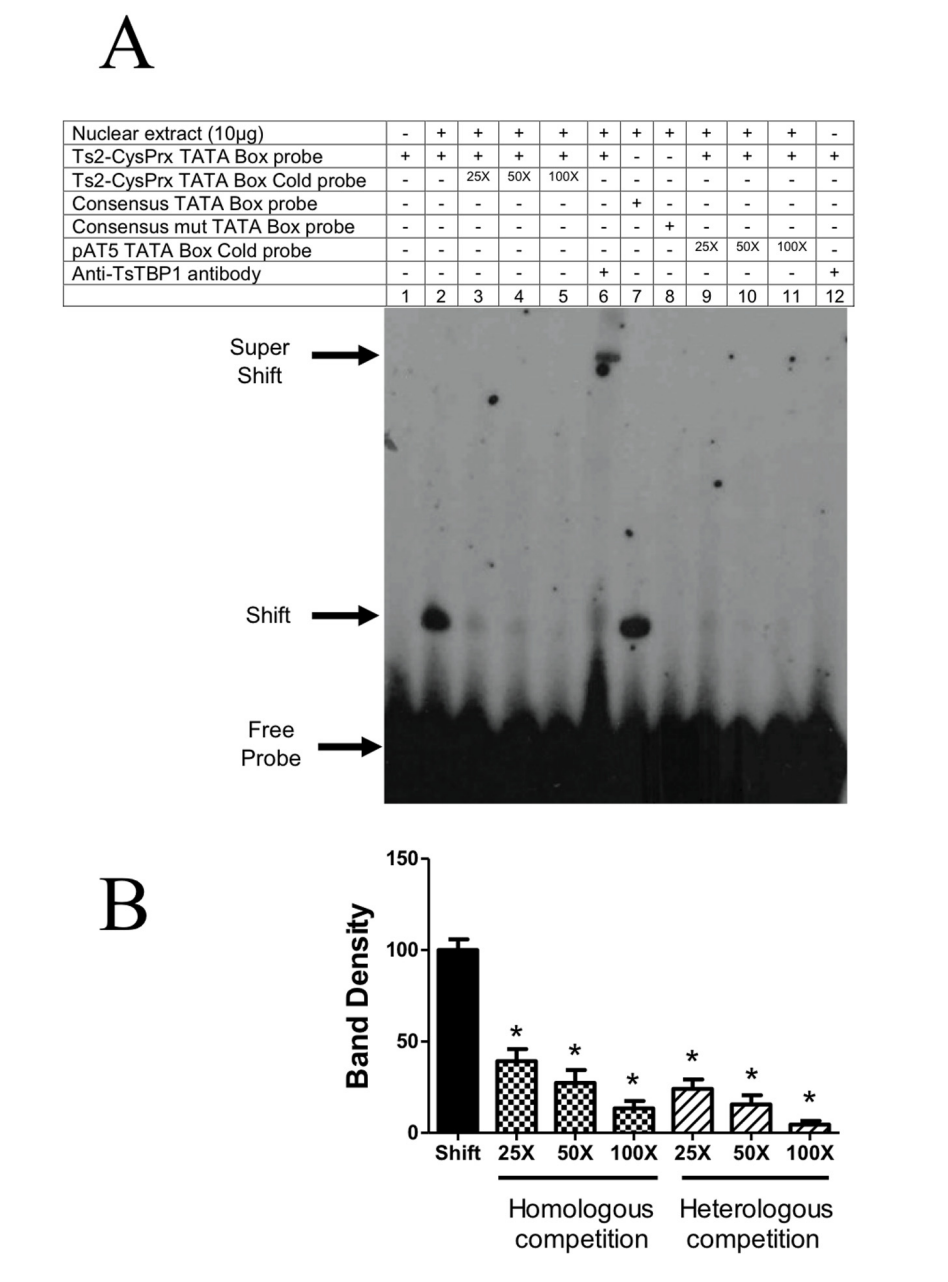
Electrophoretic mobility shift assay showing the interaction of wild type TsTBP1 with Ts2-CysPrx TATA-box probe. A) Lane 1: Labeled dsDNA-^32^P probe without nuclear extract; lane 2: TsTBP1-Ts2-CysPrx TATA-box interaction with *T*. *solium* nuclear extract; lanes 3, 4, and 5: competence with Ts2-CysPrx TATA-box cold probe in a molar excess of 25X, 50X, and 100X, respectively; lane 6: super-shift interaction using anti-pTsTBP1-N; lane 7: consensus TATA-box probe interaction with *T*. *solium* nuclear extract (used as positive control); lane 8: consensus mutated TATA-box probe interaction with nuclear extract (used as negative control); lane 9, 10, and 11: cross-competence with pAT5 TATA-box cold probe in a molar excess of 25X, 50X, and 100X, respectively; lane 12: anti-TsTBP1-N antibody without *T*. *solium* nuclear extract (negative control). Shifted, super-shifted bands, and the free-labeled dsDNA probe are indicated by arrows. B) The densitometric analysis shows a decrease on the intensity of shifted bands in homologous and heterologous competition. Results are present as percentage mean ± SD of the shifted band in lane 2 (P < 0.005).

To establish the interaction of TsTBP1 with the TATA-box motif, we performed EMSA. As DNA targets, we used two putative TATA-box motifs located in the core promoter from two *T*. *solium* genes: actin 5 (pAT5) and 2-Cys peroxiredoxin (Ts2-CysPrx), both localized between -30 to -23 bp relative to the TSS. We also used as a control the consensus TATA-box of adenovirus major late promoter (TATA-box consensus, Table 1). The interaction of TsTBP1 with the putative TATA-box of pAT5 (Fig 4) and Ts2-CysPrx (Fig 5) labeled dsDNA probes showed a shifted band produced by TsTBP1 binding to the respective probe (see lanes 2, Figs 4 and 5). In lanes 3, 4, and 5 of Fig 4A a decrease in the intensity of the shifted band can be observed due to homologous competence with TATA-box of the pAT5. Densitometric analysis shows a decrease of 17%, 72%, and 89% for 25X, 50X and 100X with the non-labeled probe, respectively (Fig 4B). In lanes 3, 4, and 5 of Fig 5A also a decrease in the intensity of shifted band were observed with TATA-box of the Ts2-CysPrx. The densitometric analysis also shows a decrease of 61%, 73% and 87% when 25X, 50X and 100X with cold probe were used, respectively (Fig 5B). In lane 6 of Figs 4 and 5, a super-shifted band is observed due to the interaction with the anti-pTsTBP1-N antibody. In lane 7 of same figures, a shifted band is also observed when the consensus TATA-box labeled probe was added to the reaction. In contrast, in lanes 8, disappearance of the shifted band is observed, when the labeled mutated consensus TATA-box probe was added.

**Table 1:** Double-stranded DNA probes used for the interaction of TsTBP1 with different TATA-box sequences by EMSA. In bold letters are represented the putative TATA-box for each gene. Underlined bases are the mutated bases in the TATA-box consensus.

pAT5 TATA-box	5’–CCCAAATCT**TATATAAA**CCGTGGGT–3’
	3’–GGGTTTAGAATATATTTGGCACCCA–5’
Ts2-CysPRX TATA-box	5’–GCGCTTCGC**TATATTTG**GCGGTAAG-3’
	3’–CGCGAAGCGATATAAACCGCCATTC–5’
Consensus TATA-box	5’–AAGGGGGGC**TATAAAAG**GGGGTGGG–3’
	3’–TTCCCCCCGATATTTTCCCCCACCC–5’
Consensus TATA-box mut	5’–AAGGGGGGC**T** **G** **T** **G** **AAAG**GGGGTGGG–3’
	3’–TTCCCCCCGATATTTTCCCCCACCC–5’

Fig 4A in lanes 9, 10, and 11, shows the competence of the interaction between TsTBP1 and the putative TATA-box of pAT5 and Ts2-CysPrx TATA-box cold probes in a molar excess of 25X, 50X, and 100X, respectively. A strong competition was seen using a 50X molar excess cold probe, where the density of the band decrease to up 36% (Fig 4B). In contrast, Fig 5A, in lanes 9, 10, and 11, shows competence of the interaction between TsTBP1 and the putative TATA-box Ts2-CysPrx with pAT5 TATA-box cold probe; a strong competition is observed starting at the 25X molar excess cold probe, where density of the band decrease to up 24% (Fig 5B). Finally, in lane 12 of Figs 4 and 5, no shifted band is observed when only anti-pTsTBP1-N antibodies were used in the reaction without nuclear extract.

### Inhibition of binding TsTBP1 to TATA-box motif

**Fig 6:**
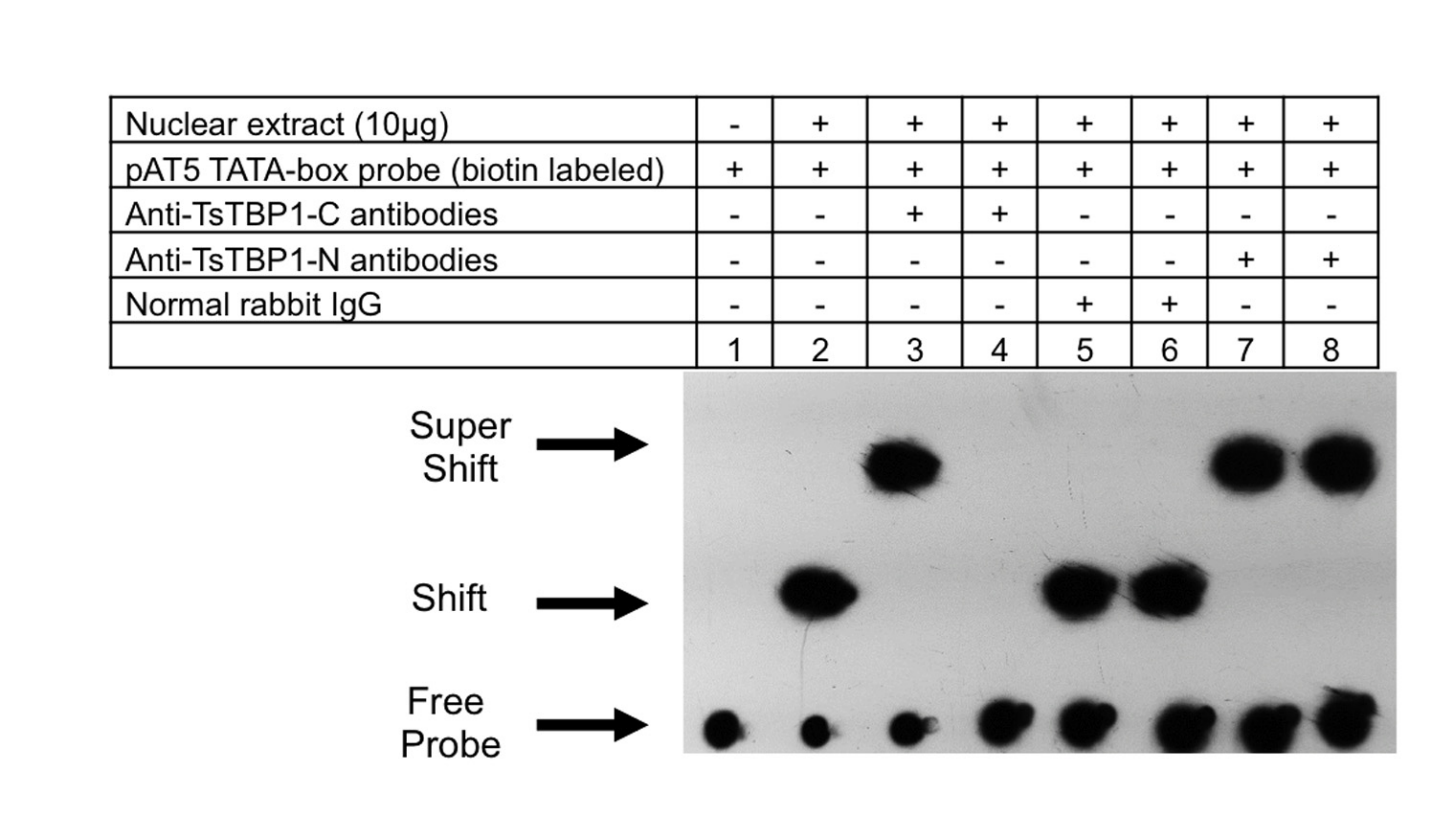
EMSA showing the inhibition of the binding of TsTBP1 to TATA-box of pAT5 by the anti-pTsTBP1-C. Lane 1: labeled TATA-box pAT5 dsDNA-biotin probe without *T*. *solium* nuclear extract; lane 2: TATA-box pAT5 interaction with *T*. *solium* nuclear extract; lane 3: TATA-box pAT5 plus *T*. *solium* nuclear extract and anti-pTsTBP1-C antibodies; lane 4: *T*. *solium* nuclear extract plus anti-pTsTBP1-C with TATA-box pAT5; lane 5: TATA-box pAT5 plus *T*. *solium* nuclear extract and normal rabbit IgG, lane 6: *T*. *solium* nuclear extract plus normal rabbit IgG and TATA box pAT5, lane 7: TATA-box pAT5 plus *T*. *solium* nuclear extract and anti-pTsTBP1-N antibodies, and lane 8: *T*. *solium* nuclear extract plus anti-pTsTBP1-N antibodies and TATA-box pAT5.

To see if antibodies were able to inhibit binding of TsTBP1 to TATA-box of pAT5, an EMSA was performed. Fig 6 shows that incubation first of *T*. *solium* nuclear extract and TATA-box of biotin-labeled pAT5, followed of the incubation with anti-pTsTBP1-C antibodies, normal rabbit IgG and anti-pTsTBP1-N antibodies, reaction produced a supershifted (lane 3), shifted (lane 5), and supershifted (lane 7) bands, respectively. In contrast, the incubation of *T*. *solium* nuclear extract with anti-pTsTBP1-C antibodies, normal rabbit IgG and anti-pTsTBP1-N antibodies, before the addition of biotin-labeled TATA-box from pAT5, produced no band (lane 4), a shifted (lane 6), and supershifted (lane 8) bands, respectively. Lane 2 shows a shifted band after *T*. *solium* nuclear extract was incubated with the biotin-labeled TATA-box probe from pAT5, and lane 1 shows no band in the biotin-labeled TATA-box probe from pAT5 in absence of nuclear extract.

## Discussion

We found two identical cDNA clones of about 900 bp, after screening about 45,000 phages of an adult *T*. *solium* cDNA library, which means that TBP1 mRNA correspond to 0.0045% of our library, this result is in agreement with reports for *Onchocerca volvulus* and for human liver, where low abundance of mRNA coding for TBP1 has been found, however is enough to maintain the PIC formation for the transcription process [37–39]. These findings suggest that the mRNA for TsTBP1 is very stable.

The amino acid deduced sequence from the cDNA phage clones and its comparison analysis with other TBPs revealed coding for TBP1 with a predicted molecular weight of 26.7 kDa, which can be divided into an NH_2_-ter and a COOH-ter. The COOH-ter is composed of 190 residues, which resulted to be highly conserved among species; moreover, it contains all amino acids involved in DNA interactions and some GTFs. The constructed homology model for TsTBP1 showed that the COOH-ter forms the classical ribbon structure composed of ~180 residues, and an extra α-helix at the end of the domain, which is composed by the amino acids SDKSGLHQPALTG. Noteworthy, the extra α-helix is also conserved in *E*. *multilocularis* and *E*. *granulosus*, but its function is unknown. The superposition of TsTBP1 structure with X-ray structures of human and yeast TBP1s shows that the recognizing motifs for DNA and GTFs (TFIIA and TFIIB) are structurally conserved in this domain. On the other hand, the model for the COOH-ter of TBP1 shows that all amino acids that interact with TATA-box are conserved and in the concave region of this domain; likewise, it shows that the amino acids that form positive patches interact with the phosphate groups and form hydrogen bonds that stabilize the TBP1-TATA-box structure. These findings indicate the relevance that the COOH-ter of TsTBP1 has in the binding of DNA and GTFs for PIC formation in the transcription process.

In contrast, the NH_2_-ter of TBP1 is composed of 48 residues and presents a high homology in length, amino acid composition, and structure with the TBP1 from *E*. *multilocularis* and *E*. *granulosus*, but only 31% identity with HsTBP1 and SsTBP1 (*T*. *solium* hosts). The constructed homology model for TsTBP1 showed that NH_2_-ter is composed of three helices connected by random coils. The NH_2_-ter domain is key for species-specific transcription factors binding and acts as a negative regulator of TBP1 function [6, 10].

Western blot assays with the anti-pTsTBP1-N and anti-pTSTBP1-C antibodies, led us to the identification of the native TsTBP1 as a 26 kDa-band in *T*. *solium* nuclear extract, this result is in agreement with the predicted molecular weight from the translated cDNA sequence of the TsTBP1 clone. The same antibodies together with anti-histone H1 antibodies and DAPI by confocal microscopy showed that TBP1 is localized in the nucleoplasm of cells of *T*. *crassiceps* cysticerci. Noteworthy, the nucleolus was observed as a dark zone with a slightly blue signal produced by DAPI, no signal was produced in this region by anti-pTsTBP1-C and anti-histone H1 antibodies, despite that histone H1 and TBP1 are components of the nucleolus. A possible explanation for this observation is the high-density produced by the rRNA transcribed in the nucleolus that does not permit the entrance of antibodies, as has been mentioned previously [40].

EMSA showed binding of TATA-box probes (adenovirus major late promoter, Ts2-CysPrx, and pAT5) to TsTBP1. This observation was further confirmed by a super-shift assay using anti-pTsTBP1-N antibodies. On the other hand, cross competition assays showed that TsTBP1 has a higher affinity for the TATA-box of pAT5 promoter than the TATA-box of Ts2-CysPrx promoter. These findings suggest that the TATA-box of the pAT5 promoter has a more stable interaction with TsTBP1 and a better formation of the PIC than the TATA-box of the Ts2-CysPrx promoter. Noteworthy, EMSA also demonstrated that anti-pTsTBP1-C antibodies recognize epitopes on the extra α-helix localized at the end of the COOH-ter of TsTBP1. These antibodies were able to inhibit the binding of TsTBP1 to the TATA-box of the pAT5 promoter; on the contrary, neither normal IgG or anti-pTsTBP1-N antibodies can inhibit this binding, which suggests that the transcription process can be disrupted in this parasite. However, more specific studies should be done to know if this inhibition is species specific.

*Echinococcus granulosus* and *T*. *solium* are parasites causing hydatidosis and neurocysticercosis in humans, diseases posing economic and health problems, and as long as governments fail to offer education and sanitary health infrastructure in developed countries, these parasites will persist [41–43]. For *T*. *solium* diseases control, there are only two drugs approved by the WHO, praziquantel, and albendazole. These drugs killing just 65% of parasites and obtaining complete cure in less than 40% of patients with neurocysticercosis [44], additionally the use of these drugs could be dangerous in patients with heavy cyst burdens, or when post- treatment inflammation causes intracranial hypertension or hydrocephalus [45]. On the other hand, *T*. *solium* has started to develop resistance against albendazole [46, 47]. For this reason is necessary more research about the design of new safe and efficacy drugs.

TBP is essential for the development of the organism and its lack leads to death [48]. Therefore, the differences found in the NH_2_- and COOH-ter between TBP1s of *Taeniidae* family and its mammalian hosts, could be used as a potential target to develop new molecules to inhibit transcription in these parasites. Monoclonal, recombinant antibodies or peptides against transcription factors could be an option, because it is known that polyclonal and monoclonal antibodies against non-conserved regions inhibit in ~74% the catalytic activity of *T*. *solium* triosephospate isomerase (TPI), and monoclonal against to *S*. *mansoni* TPI by passive immunization assays confer partial protection (41–49%) against schistosomiasis in mice [49–51]. Finally, to our knowledge, this is the first study demonstrating that TBP is involved in gene transcription of cestodes.

## Supporting Information

S1 Fig Confocal microscopy xyz planes showing fluorescence inside the nucleus. A) DAPI + TsTBP1, B) DAPI + histone H1 and C) DAPI + TsTBP1 + histone H1.
(TIF)

S2 Fig Confocal microscopy fluorescence quantification. A) Schematic representation of the fluorescence quantification and the plane used for the analysis. B) Relative abundance of histone H1 (100%) and TsTBP1 (79%), not statistical significance was observed.
(TIF)

## Abbreviations

anti-pTsTBP1-C: antibodies raised against synthetic peptide of COOH-ter
anti-pTsTBP1-N: antibodies raised against synthetic peptide of NH_2_-ter
COOH-ter: Carboxyl terminal domain
NH_2_-ter: Amino terminal domain
pAT5: *T*. *solium* actin 5 gene
PIC: Pre-initiation complex of transcription
pTsTBP1-C: peptide of COOH-ter
pTsTBP1-N: peptide of NH_2_-ter
RMSD: Root-mean-square deviation
TAF: TBP-associated factor
TBP: TATA-box binding protein
Ts2-CysPrx: *Taenia solium* 2-Cys peroxiredoxin gene
TSS: Transcription start site
TsTBP1: *Taenia solium* TATA-box binding protein 1
