# Supplementary figures and images for: Molecular Cloning of a cDNA Encoding for Taenia solium TATA-Box Binding Protein 1 (TsTBP1) and Study of Its Interactions with the TATA-Box of Actin 5 and Typical 2-Cys Peroxiredoxin Genes
Source: PLoS One. 2015 Nov 3;10(11):e0141818. doi: 10.1371/journal.pone.0141818 (PMC4631506; doi:10.1371/journal.pone.0141818)

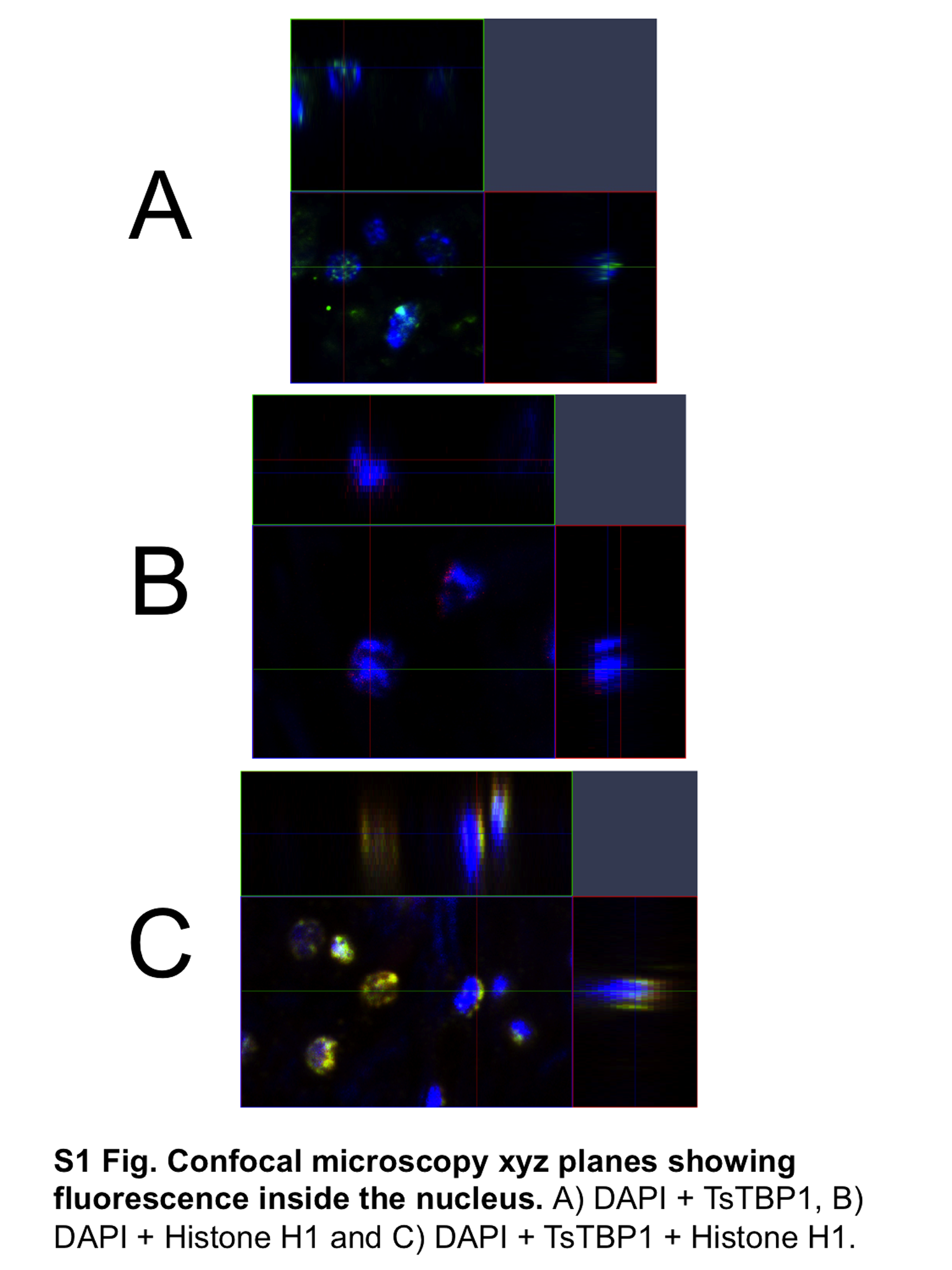

Supplement: S1 Fig — A) DAPI + TsTBP1, B) DAPI + histone H1 and C) DAPI + TsTBP1 + histone H1. (TIF) [file pone.0141818.s001.tif]

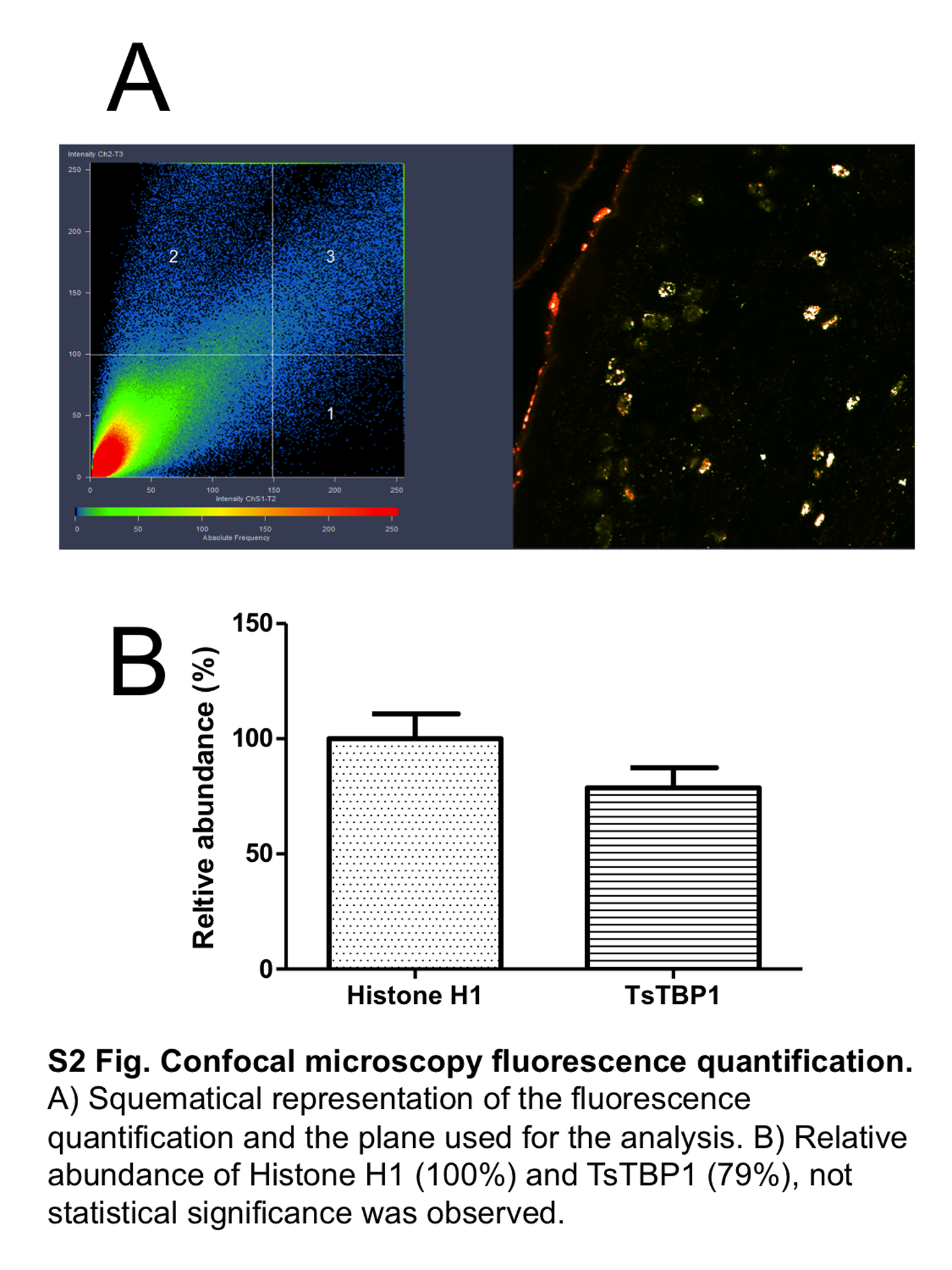

Supplement: S2 Fig — A) Schematic representation of the fluorescence quantification and the plane used for the analysis. B) Relative abundance of histone H1 (100%) and TsTBP1 (79%), not statistical significance was observed. (TIF) [file pone.0141818.s002.tif]
